# A Kunitz-type peptide from *Dendroaspis polylepis* venom as a simultaneous inhibitor of serine and cysteine proteases

**DOI:** 10.1590/1678-9199-JVATITD-2020-0037

**Published:** 2020-10-07

**Authors:** Roberto Tadashi Kodama, Alexandre Kazuo Kuniyoshi, Cristiane Castilho Fernandes da Silva, Daniela Cajado-Carvalho, Bruno Duzzi, Douglas Ceolin Mariano, Daniel C. Pimenta, Rafael Borges, Wilmar Dias da Silva, Fernanda Calheta Vieira Portaro

**Affiliations:** 1 Laboratory of Immunochemistry, Butantan Institute, São Paulo, SP, Brazil.; 2 Laboratory of Biochemistry and Biophysics, Butantan Institute, São Paulo, SP, Brazil.; 3 Department of Physics and Biophysics, Botucatu Biosciences Institute (IBB), São Paulo State University (UNESP), Botucatu, SP, Brazil.

**Keywords:** *Dendroaspis polylepis* venom, Inhibitor, Serine peptidase, Cysteine peptidase, Kunitz-type peptide

## Abstract

**Background:**

Proteases play an important role for the proper physiological functions of the most diverse organisms. When unregulated, they are associated with several pathologies. Therefore, proteases have become potential therapeutic targets regarding the search for inhibitors. Snake venoms are complex mixtures of molecules that can feature a variety of functions, including peptidase inhibition. Considering this, the present study reports the purification and characterization of a Kunitz-type peptide present in the *Dendroaspis polylepis* venom as a simultaneous inhibitor of elastase-1 and cathepsin L.

**Methods:**

The low molecular weight pool from *D. polylepis* venom was fractionated in reverse phase HPLC and all peaks were tested in fluorimetric assays. The selected fraction that presented inhibitory activity over both proteases was submitted to mass spectrometry analysis, and the obtained sequence was determined as a Kunitz-type serine protease inhibitor homolog dendrotoxin I. The molecular docking of the Kunitz peptide on the elastase was carried out in the program Z-DOCK, and the program RosettaDock was used to add hydrogens to the models, which were re-ranked using ZRANK program.

**Results:**

The fraction containing the Kunitz molecule presented similar inhibition of both elastase-1 and cathepsin L. This Kunitz-type peptide was characterized as an uncompetitive inhibitor for elastase-1, presenting an inhibition constant (K_i_) of 8 μM. The docking analysis led us to synthesize two peptides: PEP1, which was substrate for both elastase-1 and cathepsin L, and PEP2, a 30-mer cyclic peptide, which showed to be a cathepsin L competitive inhibitor, with a K_i_ of 1.96 µM, and an elastase-1 substrate.

**Conclusion:**

This work describes a Kunitz-type peptide toxin presenting inhibitory potential over serine and cysteine proteases, and this could contribute to further understand the envenomation process by *D. polylepis*. In addition, the PEP2 inhibits the cathepsin L activity with a low inhibition constant.

## Background

Snake envenomation is considered a public health problem worldwide due to its ability to cause serious accidents that can be potentially lethal if not promptly treated by the administration of specific antiophidic serum. According to World Health Organization (WHO) data, globally, around 5.4 million accidents are reported per year, causing more than 435,000 amputations and 81,000 deaths. Due to this high number of accidents, and deaths, snakebite envenomation is considered a tropical neglected disease since 2017 by the WHO [1]. On the other hand, despite its toxicity, snake venoms are also a rich mixture of bioactive compounds, which are a natural source of molecules that have been used as a benefit for human health [2, 3]. Thus, toxins can be used as drugs (or prototypes for the development of new drugs) both for their selectivity and potency. This fact can be well exemplified by bradykinin-potentiating peptides (BPPs), the first natural inhibitors described for the Angiotensin Converting Enzyme (ACE) [4]. BPPs, or proline-rich peptides, which are toxins that cause hypotension, have been used to successfully design an antihypertensive drug, captopril [5]. Because of this, and other features of the small molecules present in snake venoms, studies and research of these compounds are relevant.

*Dendroaspis polylepis* snakes, also known as black mambas, inhabit a large portion of the sub-Saharan region and their venom is considered one of the most potent among the animal kingdom. The envenomation may cause hypotension, tachycardia, paresthesia in the superior and inferior members and respiratory failure in their victims [6]. Proteomics studies revealed that the *D. polylepis* venom is mainly composed by Kunitz-type molecules, which includes mamba dendrotoxins (63%), three finger toxins (31%) and metallopeptidases (3%) [7]. The fact that this venom contains a significant number of Kunitz-type molecules, not yet characterized as peptidase inhibitors, was one of the main reasons for choosing this venom for the development of the present work.

The first Kunitz molecule was purified from soybean and was described as a potent trypsin inhibitor [8]. Later, many other inhibitors with similar properties were identified, not exclusively in plants, but also in animals and animal venoms [9, 10], and the identification of new Kunitz-type inhibitors is still being carried out. Currently, Kunitz-type molecules are classified by presenting an amino acid chain of about 60 residues, which is stabilized by three disulfide bonds, granting a characteristic fold [11]. To date, there are about 500 different Kunitz-type molecules whose sequences are deposited in the UniProt database and, although this family of inhibitors is associated with the inhibition of serine proteases, Kunitz peptides capable of inhibiting cysteine and aspartic proteases have already been described, with the exception of metallopeptidases [12]. In animal venoms, Kunitz molecules went through extraordinary functional adaptations, that is, from protease inhibitors to channel blocking neurotoxins. It is known that, during the evolutionary history of these molecules, there was a loss of protease inhibitory function and, consequently, a gain in other functions, such as sodium and potassium channel blockers [13, 14]. These new functions had independent origins in several animal phyla, including snakes [14]. A Kunitz molecule in a snake venom that presents both channel-blocking and enzyme inhibition properties had not been described until 2014, when Yang and collaborators [15] described the BF9. This was the first functionally characterized snake venom Kunitz peptide with both protease and potassium channel inhibiting properties [15]. The Kunitz molecules from snake venoms need in-depth analysis, since the presence of these peptides is more related to potassium channel inhibition activities [7] while the inhibitory properties of proteases have been little studied.

Because of their important role in the regulation of several physiological processes, proteases need to be present in adequate amounts and locations. In addition, proteases need their activities to be controlled so that the physiological processes occur in a healthy way in organisms. Thus, the activity of a protease can be controlled by factors such as pH, salt concentration, endogenous inhibitors, among others. Therefore, a small imbalance, both in the expression level and in the activity of these molecules, can contribute to the appearance of pathologies [16]. In this scenario, the search for peptidase-modulating molecules to control this activity is important.

Considering the above, this work aimed to study a Kunitz-type molecule present in the venom of *D. polylepis* that has already been described as an ion channel blocker. The toxin was selected from the low molecular weight portion of the venom by screening inhibition over the activity of the serine peptidase, elastase-1, and the cysteine peptidase, cathepsin L. This molecule was purified, sequenced, biochemically characterized, and *in silico* studies were carried out to verify the interaction of this molecule with the two enzymes cited above. From the results obtained by the *in silico* studies, two small peptides from the Kunitz-type molecule were synthesized, and both had their inhibitory properties towards the peptidases studied in this work.

## Methods

### Materials

The lyophilized crude venom of *D. polylepis* (From South Africa and Kenya) were purchased from Latoxan SAS (Valence, France). The two samples used comprised venoms from three females andtwo males, all adults (Code L1309 - *D. polylepis*, South Africa, Batch #625.011 and Code L1348 - *D. polylepis*, Kenya, Batch #525.01). The pancreatic elastase (EC 3.4.21.36) and the substrate Z-FR-MCA (used for cathepsin L assays) were purchased from Sigma-Aldrich (St Louis, MO, USA). The FRET substrate, Abz-FRSSRQ-EDDnp (used for elastase assays), was kindly provided by Prof. Dr. Luiz Juliano Neto, from the Department of Biophysics of UNIFESP-EPM. The recombinant human cathepsin L (EC 3.4.22.15) was purchased from R&D systems (EUA). The synthetic peptides were obtained by the solid-phase peptide synthesis method and purchased from GenOne Biotechnologies (Rio de Janeiro, Brazil). Their HPLC profiles and mass spectra analyses are present in Additional file 1. The purity of all peptides was analyzed by reverse-phase HPLC and the primary sequences were confirmed by analysis of MS/MS. Acetonitrile and trifluoroacetic acid (TFA) were obtained from J. T. Baker.

### Obtainment of venom peptide pools and HPLC fractionation

Lyophilized venoms (10 mg) from *D. polylepis*, from Kenya and from South Africa, were dissolved in 0.05 M ammonium acetate pH 4.2 in a final volume of 5.0 mL, and immediately filtered through a Merck Millipore Amicon Ultracel 10 K centrifugal filter device with a molecular mass cut-off of 10,000 Da (Tullagreen, Carrigtwohill, IRL), in order to prevent proteolytic cleavage of peptides by venoms. Filtrated solutions containing low molecular mass fractions were injected (500 μL) in a reverse-phase HPLC (Prominence, Shimadzu, Japan), using 0.1% trifluoroacetic acid (TFA) in water, as solvent A, and acetonitrile and solvent A (9:1) as solvent B. The separations were performed at a flow rate of 1 mL/min using a Restek Ultra C-18 column (4.6 × 150 mm) and a 20-60% gradient of solvent B over 20 min. In all cases, elution was followed by the measurement of ultraviolet absorption (214 nm). The peaks were manually collected, dried and subjected to enzymatic assays.

### Searching for elastase-1 and cathepsin L inhibitors

Elastase enzymatic assays were conducted in Tris HCl 50mM, NaCl 50 mM, pH 8.0 and, for cathepsin L, the buffer used was sodium phosphate 50 mM, NaCl 200 mM, EDTA 5 mM, DTT 6.25 mM, pH 5.5.The reactions were initiated by the addition of elastase-1 (100 ng) or cathepsin L (1 ng), using the substrates Abz-FRSSRQ-EDDnp (5 μM) and Z-FR-MCA (5 μM), respectively. The enzymatic reactions were performed in a fluorimeter (Hidex, Hidex sense 425-301, Finland), using 96-well plates (final volume 100 μL), adjusted for excitation and emission readings at 320 and 420 nm, respectively, for the FRET substrate, and excitation and emission at 360 nm 480 nm, respectively, for the substrate Z-FR-MCA. The peptidases inhibition assays were performed using 20 μL of each collected peak, obtained from the HPLC fractionations. The temperature remained constant at 37 °C and one reading per minute was performed for 15 minutes, the plates being shaken before each measurement. All assays were performed in duplicate, and the specific activities were expressed as units of free fluorescence of the cleaved substrates per μg of peptidase per min (UF/μg/min). The fractions that showed inhibition greater than 50% were analyzed by mass spectrometry.

### Identification of the content present in the inhibitory fractions by MS/MS

The selected fractions were dried and resuspended in 10 μL of 0.1% trifluoroacetic acid. Each sample (1 μL) was co-crystallized with alpha-cyano-4-hydroxycinnamic acid (saturated solution in acetonitrile/water/0.1% TFA) (matrix), placed in sampler for drying and analyzed in a MALDI-TOF/TOF spectrometer (Axima Performance, Shimadzu). Spectra were obtained using the positive linear mode and a mass interval between 50 - 13,000 mass/charge (m/z). To sequence the peptides, the content of each fraction was also subjected to a trypsin digestion (in solution), as described [17]. Prior to the mass spectrometer analysis, the samples were desalted (ZipTip® C-18 pipette tips, Millipore) and dried. Each sample was resuspended in 30 µL of 0.1% formic acid, and the LC-MS/MS analyses were carried out in the LTQ Orbitrap Velos (ThermoScientific). The fractions were then automatically injected into a 5 cm C-18 pre-column packed with Jupiter 10 μm resin (Phenomenex; 100 μm I.D.) using the Easy-nLC II system (Thermo Scientific). After the loading process, the peptides were subjected to a chromatographic separation in a 10 cm C-18 column packed with AQUA 5 μm resin (Phenomenex; 75 μm I.D.) at a constant flow rate of 200 nL/min. The peptides were separated with a gradient of 5-15% B (B: 0.1% formic acid in acetonitrile) in 10 min; 15-35% B in 30 min; 35-85% B in 5 min; 85-5% B in 2 min and 5% B in 8 min. The MS and MS/MS spectra were acquired by the Orbitrap module (*scan range*: 400-2000 m/z; full scan resolution: 30,000; scan resolution of MS/MS: 7,500). The eluate was electro-sprayed at + 1.8 kV and the instrument was operated on Data Dependent Acquisition (DDA), where the ten most intense ions per scan were selected for fragmentation by HCD (higher*-*energy collisional dissociation). The dynamic exclusion time used was 90 s repeating in intervals of 30 s. The MS and MS/MS spectra were submitted to bioinformatics analyses using the PEAKS Studio 8.5 software (Bioinformatics Solutions Inc.). *De novo* analyses were performed with tolerances of 15 ppm and 0.025 Da for precursor and fragment ions, respectively. Methionine oxidation, carbamidomethylation and deamidation were considered variable modifications. The obtained sequences were confronted with the “animal toxins annotation project” UniProt data available inhttps://www.uniprot.org/program/Toxins.

### Characterization of Kunitz-type peptide from *D. polylepis* peptide pool as elastase-1 inhibitor

To determine the inhibition constant (K_*i*_ ) of the peptide, firstly the Michaelis-Menten constant (K_*m*_ ) for the Abz-FRSSRQ-EDDnp hydrolysis by elastase-1 was obtained. For this, the concentrations of 2.5 µM, 5.0 µM, 10.0 µM, 15.0 µM, 25.0 µM and 50.0 µM of the substrate were used, in Tris 50 mM, NaCl 50 mM pH 8.0. Initial rates were determined at an enzyme concentration (100 ng) that would hydrolyze less than 10% of the substrate. The kinetic parameters (K_*m*_ and V_*max*_ ) were calculated by Michaelis-Menten equation using the Grafit 5 software (Erithacus Software, West Sussex, UK), as previously described [18]. All experiments were performed in duplicate.

The Kunitz-type peptide quantification was determined by the BCA method [19] using insulin as standard (5 µg/µL, 10 µg/µL, 25 µg/µL, 50 µg/µL e 100 µg/µL). To determine the mechanism of action of the Kunitz-type inhibitor, two concentrations of Abz-FRSSRQ-EDDnp, 6.8 µM and 3.4 µM, and two concentrations of the purified peptide, 0.63 µM and 0.94 µM, were utilized. For each substrate concentration it was made a control test (absence of the peptide). The assays were performed in duplicate and during all experiment the consumption of substrate was maintained under 10%. The reactions were monitored in the fluorimeter, as described above, and were analyzed in the Grafit 5 software (Erithacus Software, West Sussex, UK). The mechanism and the inhibition constants were determined through the Dixon plot (1/V x [I]) equation [20]. The plot was made in the software GraphPad Prism 5.

### Docking analysis and prediction of the interaction of the Kunitz-type peptide and elastase-1

For these analyses, the coordinates of elastase-1 in complex with a Kunitz-type molecule (mutant ShPI-1 Lys13Leu) were obtained from the Protein Data Bank (PDB ID: 3UOU; resolution 2.00 Å) and used as template. The molecular docking was performed using the program Z-DOCK [21]. The binder models were also obtained from the Protein Data Bank (PDB ID: 1DEN, 29 RMN models). Among these models, 26 were selected due to the presence of a larger variance among each other, and measurements were made by the RMSD. For each model, 2,000 solutions were predicted, generating a total of 52,000 models. The addition of hydrogens in these models was made using the RosettaDock program, and the 52,000 model were re-ranked by the ZRANK program. This methodology was adapted from Pierce and Weng [22]. The contact surface of the theoretical complex, and amino acid residues interactions in the intermolecular interface of the models that indicated the better interactions between the ligand-receptor, were assessed with LigPlot + version 1.4 [23]. The models were visualized using PyMOL Molecular Graphics System, Version 2.0 Schrödinger, LLC.

### Stability test of the synthetic peptides

The synthetic peptides PEP1 and PEP2 (50 mM) were incubated with elastase-1 (300 ng), in Tris 50 mM, NaCl 50 mM pH 8.0 buffer, and cathepsin L (10 ng), in 50 mM, NaCl 200 mM, EDTA 5 mM, DTT 6.25 mM pH 5.5 buffer, at 37 ^o^C in two different time intervals, 4 hours and overnight. Samples containing only the synthetic peptides were used as negative control. After incubation, samples were analyzed, in duplicate by reverse phase chromatography on HPLC, using a Shim-pack C-18 column in the same conditions described above.

### Characterization of PEP2 as cathepsin L inhibitor

To obtain the Michaelis-Menten constant (K_*m*_ ) of the Z-FR-MCA hydrolysis by cathepsin L (1 ng), 1 µM, 2.5 µM, 5.0 µM, 7.5 µM, 12.5 µM, 15 µM, 20 µM concentrations of Z-FR-MCA substrate were tested in 50 mM, NaCl 200 mM, EDTA 5 mM, DTT 6.25 mM pH 5.5 buffer (final volume of 100 µL).The reactions were continuously monitored (fluorescence at λ_EM_ 480 nm and λ_EX_ 360 nm) in a fluorimeter (425-301 Hidex, Finland), as described. The maximum speed (V_*max*_ ) and the K_*m*_ were obtained in the Grafit 5 software. All experiments were performed in duplicate. To determine the mechanism of inhibition and the inhibition constant (K_*i*_ ) of inhibitor PEP2, two concentrations of the Z-FR-MCA, 5.2 µM (2 K_*m*_ ) and 7.8 (3 K_*m*_ ), and three concentrations of the inhibitor PEP2, 1 µM, 2.5 µM and 5 µM, were used. Samples without PEP2 for each substrate concentration were utilized. The experiments were carried out in duplicates and the consumption of the substrate was maintained under 10%. The results were analyzed in the Grafit 5 software. The mechanism and the inhibition constants were determined through the Dixon plot (1/V x [I]) equation [20], using the software GraphPad Prism 5.

## Results

### Fractionation of *D. polylepis* venoms

**Figure 1.**
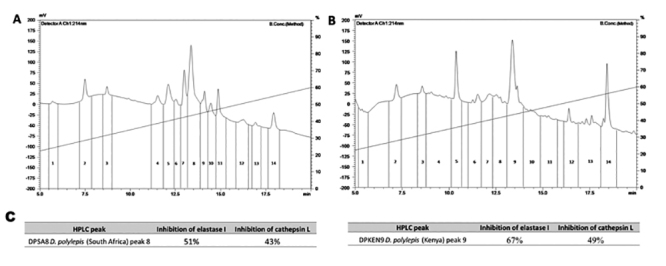
C18-LC-HPLC chromatographic profile and fractions collected from the peptide pool from the venom of *Dendroaspis polylepis* from **(A)** South Africa and from **(B)** Kenya. The blue line represents the concentration of B solution, flow: 1 mL/min. **(C)** Percentage of inhibition of catalytic activities of elastase-1 and cathepsin L by the fractions DPSA8 and DPKEN9 (20 µL),

The RP-HPLC separation of the low molecular mass constituents (< 10 kDa) from the venom of *D. polylepis* (from South Africa and Kenya) showed the profile displayed in the Figure 1 (panels A and B, respectively). The peaks were manually collected and screened using Z-FR-MCA and Abz-FRSSRQ-EDDnp as substrates to select the fractions of interest that were able to inhibit both cathepsin L and elastase-1, respectively.

**Figure 2.**
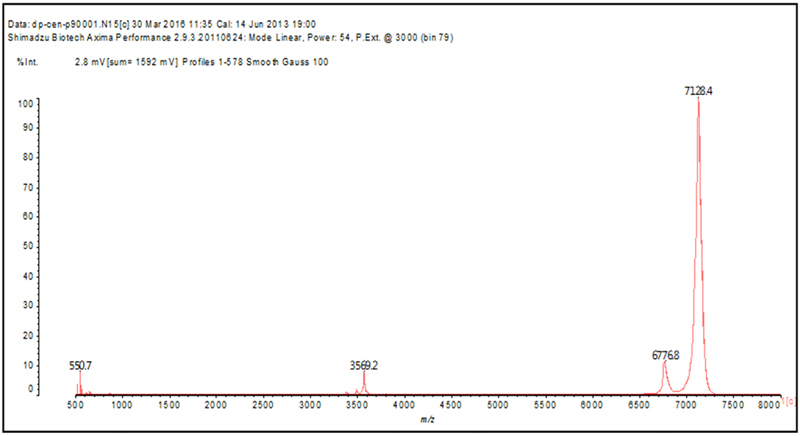
MALDI-TOF spectra (m/z) of the content present in the sample DPKEN9.

Chromatographic profiles of both *D. polylepis* venoms were relatively similar, but not identical, as there are differences in intensity, and some peaks are unique to each venom sample. The peak number 8 of *D. polylepis* venom from South Africa (DPSA8) and number 9 of *D. polylepis* from Kenya (DPKEN9) presented close retention time on C18 column and showed similar inhibition in the screening process, both for elastase-1 and for cathepsin L. In both cases, the inhibition was approximately 50% (Figure 1, panel C) and, therefore, the DPSA8 and DPKEN9 peaks were analyzed by MALDI-TOF and LTQ Orbitrap mass spectrometry. The MALDI-TOF spectra results were equal for both samples, showing the existence of one major peak with 7128.4 Da (Figure 2).

### Identification and coverage of DPSA8

**Figure 3.**
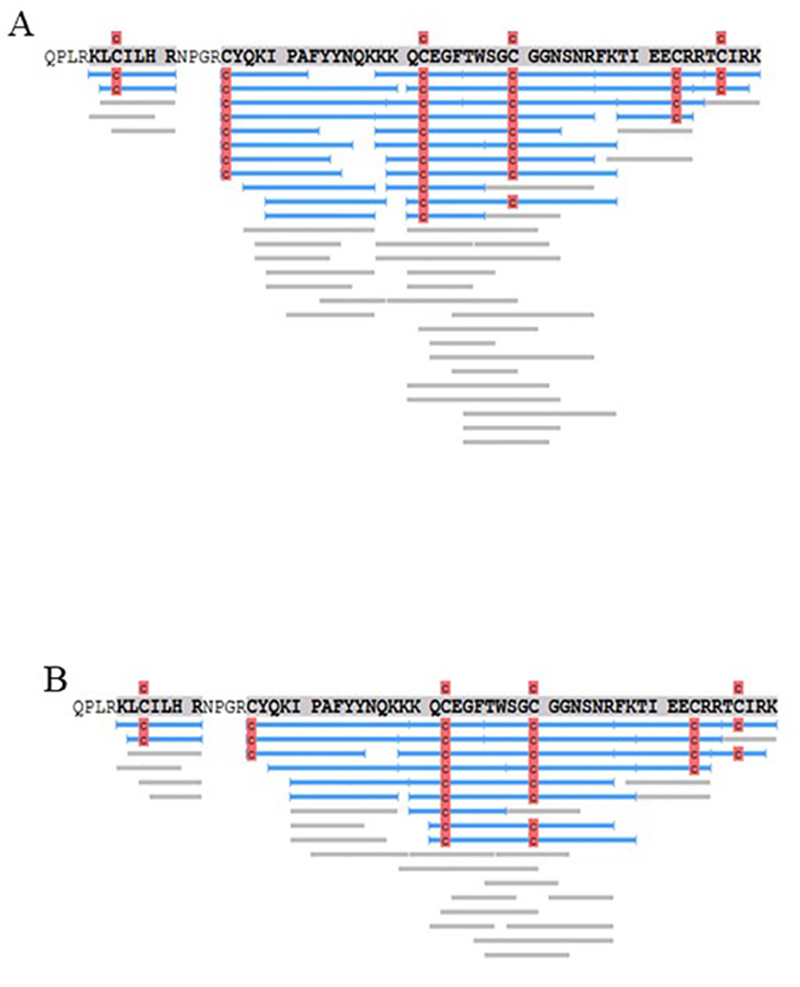
Sequences of the molecule present in the samples named **(A)** DPSA8 and **(B)** DPKEN9. The samples were subjected to a tryptic digestion and the LTQ Orbitrap results of the digested fragments were confronted with the database. In blue, peptides found with their respective modifications, in gray, the sequences of the *de novo* only with 100% of coverage, in red the carbamidomethylationof the cysteine residues. C: carbamidomethylation (+57.02). The results showed that the molecule is a Kunitz-type serine protease inhibitor homolog dendrotoxin I (Uniprot ID P00979).

The Orbitrap/PEAKS analysis of DPSA8 and DPKEN9 resulted in identification of an already described dendrotoxin from the *D. polylepis* venom (P00979), showing a coverage of 87% of its primary sequence for analyses of both samples (Figure 3, panels A and B, respectively). All peptide fragments are shown in Additional file 2 and Additional file 3, respectively. As expected, both peaks, DPKEN9 and DPSA8, contains the same molecule.

This molecule was described by Robertson and collaborators [24] in the venom of *D. polylepis* as a Kunitz-type serine protease inhibitor homolog dendrotoxin I (Uniprot ID P00979) with potassium channel blocker activity (IC_50_ = 0.13-50 nM). However, this peptide has not yet been described as a peptidase inhibitor.

### Kunitz-type peptide as an inhibitor of elastase-1

Continuing the characterization of the peptide, its inhibition properties on the elastase-1 activity were performed. Before starting the kinetic analysis with the Kunitz-like peptide and elastase-1, the concentration range of the peptide to be used was determined. For this purpose, the inhibitory effect of the peptide at concentrations of 0.63 µM, 0.94 µM, 1.26 µM and 3.52 µM was analyzed. Since the Kunitz-like peptide doses of 1.26 µM and 3.52 µM resulted in elastase inhibitions above 70% (data not shown), concentrations of 0.63 µM and 0.94 were used to determine the K*i* value and the inhibition mechanism of the peptide. After, it was necessary to determine the Michaelis-Menten constant (K_*m*_ ) of the Abz-FRSSRQ-EDDnp substrate cleavage by the elastase-1. The experimental procedure was described in the methodology section, and the K_*m*_ was settled as 10.65 µM (Additional file 4).

**Figure 4.**
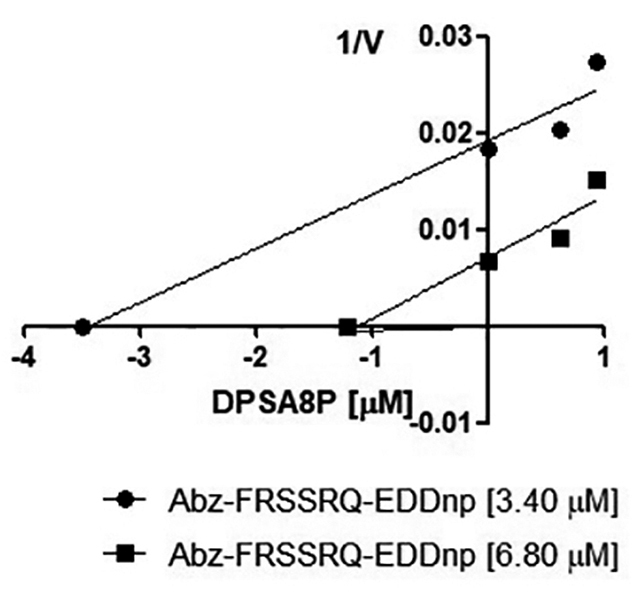
Dixon Plot built with the results of the inhibition assay of the elastase-1 activity over the FRET substrate Abz-FRSSRQ-EDDnp (3.40 and 6.80 µM), in the presence of the peptide (0.63 and 0.94 µM). The parallel lines indicate that there is the uncompetitive mechanism of inhibition. The inhibition constant was set as 8 µM. The graphic was made in the software GraphPad Prism 5.

To determine the inhibition mechanism of the Kunitz-type peptide over the elastase-1, fluorimetric assays were carried out as described in the methodology section. It was drawn curves on a Dixon plot (1/V versus [I]) (Figure 4), and the mechanism of inhibition was determined as uncompetitive inhibitor. The K_*i*_ value was calculated by the intercept of the trend lines with the abscissa axis, following the equation -K_*i*_ = (1 + K_*m*_ / [S]) [20]. Thus, the value of the inhibition constant obtained was K_*i*_ = 8 µM. 

### Molecular docking of the Kunitz-type peptide with elastase-1

**Figure 5.**
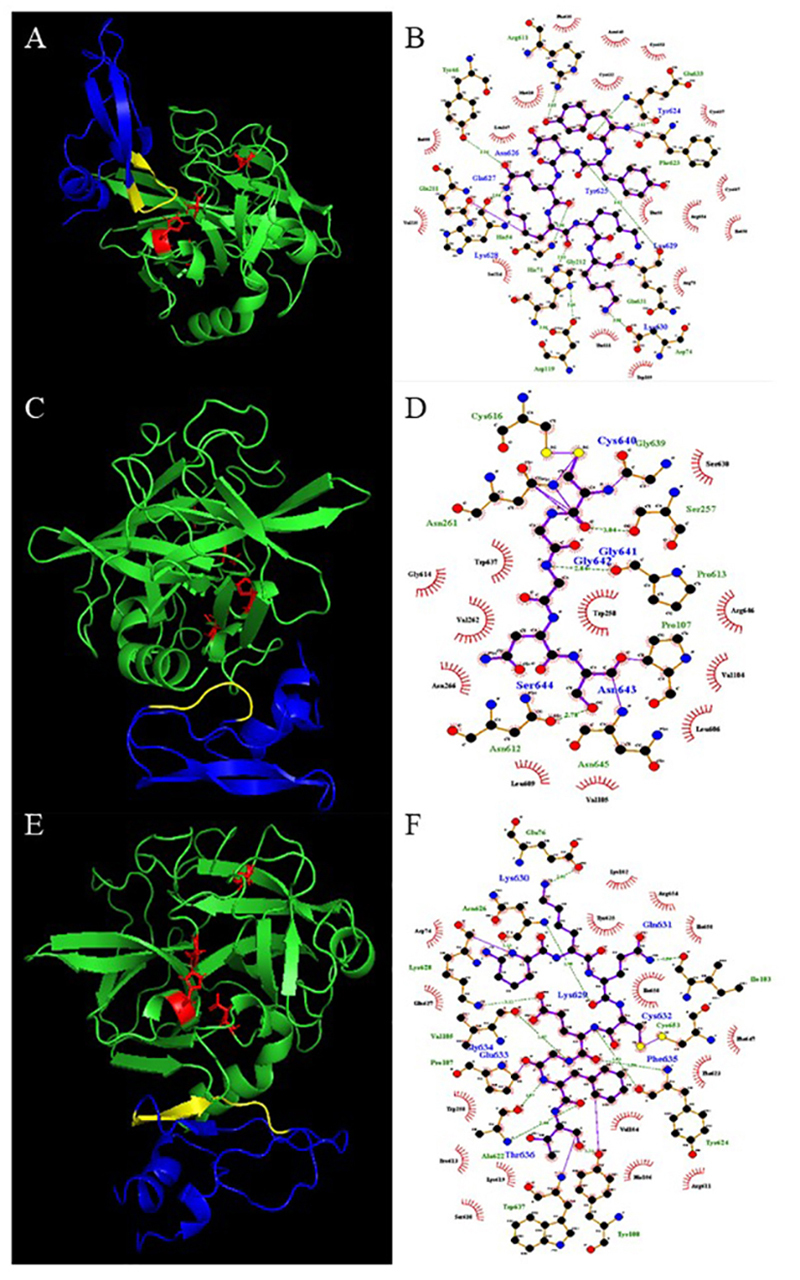
**(A)** Z-DOCK 3D model, Kunitz-type peptide in blue (YNQKKKQ region in yellow), elastase-1 in green and the amino acid residues of the catalytic triad plus the S^214^ in red. **(B)** LigPlot 2D representation of the interactions of the YNQKKKQ region of the peptide and peptidase. **(C)** z-dock 3D model, peptide in blue (CGGNS region in yellow), elastase-1 in green and the amino acid residues of the catalytic triad, plus the S^214^ in red. **(D)** LigPlot 2D representation of the interactions of the CGGNS region of the peptide and peptidase. **(E)** Z-DOCK 3D model, peptide in blue (KKQCEGFT region in yellow), elastase-1 in green and the amino acid residues of the catalytic triad plus the S^214^ in red. **(F)** LigPlot 2D representation of the interactions of the KKQCEGFT region of the peptide and peptidase.

Considering the results of inhibition, it was concluded that there is interaction between the peptide and elastase-1. Taking this into account, molecular docking analyses between these two molecules were carried out. It is also important to point that this peptide is not an elastase-1 substrate, since this molecule showed to be stable even after an overnight incubation (data not shown). The results are in accordance with the inhibition mechanism described, since that from the 40 models with the highest scores obtained, only two showed interaction of Kunitz-type peptide with the catalytic site of elastase-1, which includes the model with the highest score. The Figure 5 shows the predicted models with interaction in the catalytic site (Figure 5, panels A and B), showing the interactions of the amino acids His71, Asp119 and Ser214, that compose the catalytic triad of the peptidase, and the residues 25 to 31 of the peptide, which comprises the sequence YNQKKKQ. The Figure 5 (panels C to F), shows the interaction of the peptide with elastase-1 in regions that are not the catalytic site of the peptidase. Both interactions are close to the alpha-helix of the C-terminal region of the peptidase. In these models (panels C and D) it is possible to see the amino acids 40 to 44 (CGGNS sequence) of the peptide near to the alpha-helix mentioned above. The Figure 5 (panels E and F) indicates an interaction of the peptide with a region of elastase-1 near to the observed in the panels C and D, however, in this model, the peptide region that interacts with elastase-1 comprises the amino acid residues 29 to 36 (KKQCEGFT sequence).

### Synthesis of the peptides

**Figure 6.**
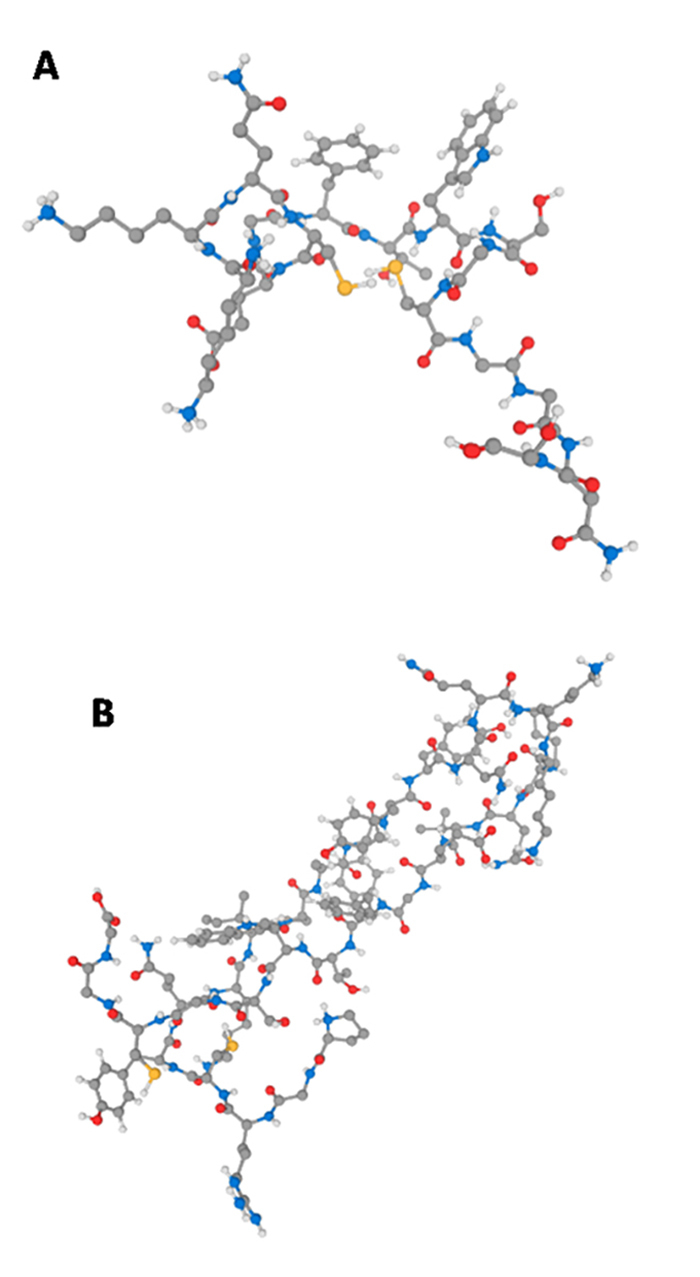
**(A)** Predicted 3D model of PEP1 (KKQCEGFTWSGCGGNS) in the PEP-FOLD server, carbon in gray, hydrogen in white, nitrogen in blue, oxygen in red and sulfur in yellow. **(B)** Predicted 3D model of PEP1 (PGRCYQKIPAFYYNQKKKQVEGFTWSGCGG) in the PEP-FOLD server, carbon in gray, hydrogen in white, nitrogen in blue, oxygen in red and sulfur in yellow.

Based on the results of docking analysis, two peptides were synthesized, minding the interaction presented in the models to create a rational design of each peptide. The first peptide, named PEP1, was designed using the data from the models that showed no interaction of Kunitz-type peptide with the catalytic site. The sequence of PEP1 is KKQCEGFTWSGCGGNS, which comprises the amino acids 29 to 44 from the peptide (the underlined amino acid residues are the ones represented in yellow in the Figure 5, panels C to F). This peptide is constituted by two different interacting regions of peptide according to the models: the fragment KKQCEGFT (Figure 5, panels E and F, amino acid residues 29 to 36) and CGGNS (Figure 5, panels C and D, amino acid residues 40 to 44), connected by the tripeptide WSG (original from the Kunitz-type molecule). This peptide is composed by 16 amino acid residues and molecular mass of 1689.01 Da, theoretical pI of 8.05 and is considered stable, according to the instability index (value of 12.48). The value of the GRAVY index is -0,956 which means that this peptide is a hydrophilic peptide. The structure prediction made by PEP-FOLD software (Figure 6) showed that this peptide is disposed in a linear structure.

The PEP2, which has the sequence PGRCYQKIPAFYYNQKKKQVEGFTWSGCGG, is made up the tripeptide CGG, from the sequence CGGNS (Figure 5, panels C and D, amino acid residues 40 to 44). The PEP2 also contains the YNQKKKQ sequence (amino acids in peptide positions 25 to 31), and this fragment corresponds to the peptide region that interacted with the catalytic site of elastase-1. The KKQVEGFT sequence (amino acids 29 to 36) is also present in the PEP2 and, in the peptide, the original sequence is KKQCEGFT, but the residue Cys of this fragment was substituted by a residue of Val. The modification aimed to design a cyclic peptide, but with a disulfide bond between the C- and N-terminal Cys residues (Cys4-Cys28). For this, the PGRCYQKIPAFY sequence was incorporated in the PEP2, enabling the formation of a disulfide bond and exposing the region that contains the YNQKKKQ sequence to interact with the active site of the elastase-1. In addition, Val is a preferred amino acid at position P1 for substrates of elastase-1 and position P3 for substrates of cathepsin L [25]. PEP2 is composed by 30 amino acid residues, presenting a molecular weight of 3,439.95 Da, theoretical pI is 9.46, and it is considered stable, according to the instability index (value of 29.25). The value of the GRAVY index is -0.933 which means that this peptide is hydrophilic. The structure prediction made by PEP-FOLD software (Figure 6B) showed that this peptide is disposed in a folded structure that expose the fragment YNQKKKQ.

**Figure 7.**
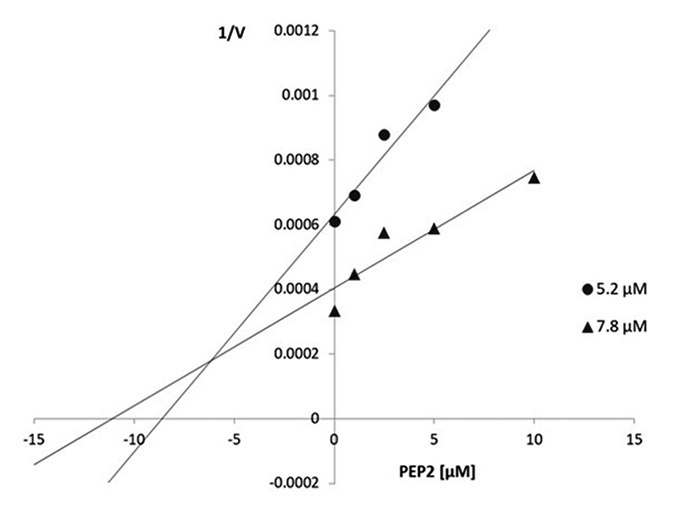
Dixon plot graphic of the inhibition of the cathepsin L activity over the Z-FR-MCA (5.2 and 7.8 µM) by the peptide PEP2 (0; 1.0; 2.5 and 5.0 µM). The intercept in the second quadrant indicates the competitive mechanism of inhibition. The inhibition constant has the value of 1.96 µM.

### Stability of PEP1 and PEP2 against hydrolysis by elastase-1 and cathepsin L

The stability of synthetic peptides, PEP1 and PEP2, was evaluated as described in the methodology. The integrity of both was assessed by C18-RP-HPLC chromatographic profiles and, both PEP1 and PEP2 were not stable under the elastase-1 activity. The PEP1 was also hydrolyzed by cathepsin L; however, the PEP2 was not degraded in incubation of 4 and 16 hours (data do not show).

### PEP2 as a cathepsin L inhibitor

For the biochemical characterization of the PEP2 as an inhibitor of cathepsin L, it was necessary to determine the Michaelis-Menten constant (K_*m*_ ) of the Z-FR-MCA substrate for cathepsin L hydrolysis, which was set as 2.65 µM (Additional file 4). The mechanism of inhibition as well as the inhibition constant of PEP2 were determined performing fluorimetric assays, utilizing 4 different concentrations of the inhibitor and two different concentrations of the substrate (2 and 3 K_*m*_ ) Z-FR-MCA. The velocities were obtained and used to construct the Dixon plot (1/V *vs* [I]). The results are shown in the Figure 7, and the line intersections in the second quadrant indicates that the mechanism of inhibition is competitive, and the value of the inhibition constant was set as 1.96 µM.

## Discussion

Snakes from the *D. polylepis* species inhabit mainly the central, east, west and south regions of the sub-Saharan Africa region [26]. This species lives in the savannahs, forests and among the rocks. It feeds on small mammals as rats, squirrels and bats, as well as small birds. Considering that most of the venom of *D. polylepis* is composed by low molecular mass peptides [7], this study aimed to verify the inhibitory property of the peptide portion of this venom upon two medically important peptidases.

In this work, *D. polylepis* venoms from two African countries were used: South Africa and Kenya. Although the chromatographic profiles of the low molecular weight pools of the two venoms show similarities, differences between the signal intensities, and exclusive components of each sample, were observed. One of the factors that may explain these differences is the geographical distance between South Africa and Kenya, which is about 4000 km apart. In fact, differences between venom compositions from the same species of snake, but from different locations, have already been reported. There are examples of intraspecific variation in snake venom between *Crotalus scutulatus* venoms from two populations, Arizona and California - USA, which showed a notable difference in the LD_50_ value when injected into rats [27]. Another example is the difference in HPLC profiles, transcriptomic and proteomic analyzes of venoms from populations of *Bothrops jararaca* collected in the southwest and south of the Brazilian Atlantic forest [28].

Regarding the similarities of the samples studied in this work, it is clear that the retention time and signal intensity on the HPLC profile are similar for the peaks 2, 3 and 4. In addition to these, other peaks that stood out in the issue of similarity were the peaks DPSA8 (South Africa) and DPKEN9 (Kenya), both in signal intensity and retention time. In fact, the similarities for these peaks go beyond the HPLC profile, since these fractions were able to inhibit the elastase-1 and cathepsin L in a very near percentage in the initial screening. Finally, it was observed a peptide with the same molecular mass in both peaks, and the primary sequence analyzes confirmed the presence of the peptide, called “Kunitz-type serine protease inhibitor homolog dendrotoxin I” (Uniprot ID P00979), with coverage above 85%. These results indicate that this molecule is preserved in *D. polylepis* venom, despite the geographic distance of the specimens where the venom came from. This fact led us to consider the importance of this molecule in the *D. polylepis* venom.

The P00979 tridimensional structure was elucidated by NMR analysis [29] and, after, this peptide was described as a dendrotoxin, with a potent potassium channel blocking action (IC_50_ = 0.13-50 nM), but its possible protease inhibitory activity has not been investigated [24]. It is worth to say that dendrotoxins are a class of presynaptic neurotoxins present in the venom of snakes of the *Dendroaspis* genus, and that is why these toxins are called this way. Despite being largely studied as potassium channel blockers in neurons, dendrotoxins have high structural homology to Kunitz-type inhibitors. Thus, the high structural similarity between dendrotoxins and Kunitz-type inhibitors is related to the evolutionary history of these molecules, some of which have lost the function of protease inhibition with consequent gain in the ability to perform the function of ion channel blockers [13, 14]. These new functions had their origin in the several animals, including the order Squamata class, and, more specifically, in snakes [14]. Just recently a Kunitz peptide was described as able to inhibit a peptidase, K_*i*_ of 18 nM for chymotrypsin, and as a potassium channel blocker, Kv1.3, IC_50_ of 120 nM [15], simultaneously.

Although the Kunitz-type peptide has already been described as able to block ionic channels [24], this work describes for the first time that this peptide is also able to inhibit proteases from different classes, elastase-1 and cathepsin L. Regarding this double inhibitory activity, it is worth mentioning that Kunitz type inhibitors capable of inhibiting cysteine proteases have been described [30, 31], but not serine proteases simultaneously. The kinetic analyses of this peptide as an elastase-1 inhibitor showed that the peptide has a K_*i*_ of 8 µM, and an uncompetitive mechanism of inhibition. When the K_*i*_ value obtained in elastase-1 assays is compared with others from Kunitz molecules present in animal venoms, the potency of the peptide here described is lower concerning the inhibition of serine peptidases. For instance, there is a Kunitz molecule, purified from sea anemones, that has the inhibition constant in the order of nM for the elastase-1 inhibition [32]. It is important to note that no other serine protease inhibitions, such as trypsin and chymotrypsin, were observed (data not shown). The uncompetitive inhibition mechanism for elastase-1 inhibition is unusual, since only Kunitz molecules have been described that have the competitive mechanism [33], as well as Kunitz with non-competitive mechanism [34, 35]. Hence, this is the first time that a Kunitz-type molecule is described as an uncompetitive inhibitor. 

The fact that Kunitz-type peptide presents inhibition of serine and cysteine peptidases, and was described as voltage-gated potassium channels blocker was unexpected, and led us to verify the interaction of this molecule via docking analyses. Considering the results, two models presented interaction with regions different than the elastase-1 catalytic site, which is in accordance with the determined uncompetitive mechanism of inhibition. The third model showed an interaction of a peptide region, amino acids 25 to 31 and sequence YNQKKKQ, with the catalytic site of elastase-1. Despite this result, the preferred amino acid residues for the interaction of elastase-1 with the substrate are not observed in this portion of the peptide, that is, alanine or valine residues in the P1 position. On the other hand, the presence of three lysine residues draws attention, as they can interact with the subsites S2, S1 and S3 of the elastase-1 [25].

Considering the results and the exposed above, two peptides were synthesized, peptides PEP1 and PEP2. The PEP1 is a linear peptide with sequence KKQCEGFTWSGCGGNS, which is homologous to amino acid residues 29 to 44 of the Kunitz peptide, and was not stable towards the elastase-1 and cathepsin L. PEP2, which has the sequence PGRCYQKIPAFYYNQKKKQVEGFTWSGCGG, is a cyclic peptide consisting of 30 amino acid residues and has the same composition as amino acid residues 13 to 42 of the Kunitz peptide, with the exception of cysteine 32, which has been replaced by a valine residue.

Although PEP2 was a substrate for the elastase-1, it showed to be stable towards cathepsin L activity. Even more relevant, this peptide was determined as a competitive inhibitor of cathepsin L, with a K_i_ value of 1.96 µM. Even though this peptide is a competitive inhibitor it is not possible to say that PEP2 binds to the catalytic site of the cathepsin L, as there are diverse feasible molecular mechanisms by which a protease may bind either the inhibitor or the substrate, but never both at the same time. The fact that the PEP2 is a good inhibitor of the cathepsin L shows that is possible to synthesize fragments of a Kunitz-type molecule that maintain the inhibitory capacity. *Ex vivo* studies are being carried out to verify the potential of PEP2 as a cathepsin L inhibitor in tumor cell culture.

## Conclusion

In this work, the inhibition of elastase-1 and cathepsin L by a Kunitz-type peptide (Uniprot ID P00979) present in the low molecular mass portion of the *D. polylepis* venom was demonstrated. This peptide had previously been described as a dendrotoxin, indicating that other molecules of this toxin class may have a dual function: potassium channel blocker and protease inhibitor. The molecular docking analyzes of Kunitz peptide with elastase-1, together with knowledge about the primary specificity of cathepsin L, served as the basis for the rational design of PEP2, and this proved to be an efficient inhibitor of cathepsin L, with a K_*i*_ of 1.96 μM. Thus, toxins present in snake venoms have great potential to serve as a basis for the design of new molecules with potential biotechnological application.

### Abbreviations

Abz: *o*-aminobenzoic acid; ACE: angiotensin converting enzyme; BCA: bicinchoninic acid; BPPs: bradykinin-potentiating peptides; CHCA: alpha-cyano-4-hydroxycinnamic acid; Da: Dalton; DTT: dithiothreitol; EDDnp: *N*-(2,4-dinitrophenyl)-ethylenediamine; EDTA: ethylene diamine tetraacetic acid; HPLC: high performance liquid chromatography; K_*i*_: inhibition constant; K_*m*_: Michaelis-Menten constant; kV: kilovolt; LTQ: linear trap quadropole; m/z line: mass-to-charge ratio; MALDI-TOF/TOF: matrix assisted laser desorption ionization time of flight mass spectrometry; MCA: 7-amino-4-methyl-coumarin; MS/MS: tandem mass spectrometry; MS: mass spectrometric or mass spectrometry; nLC: n*ano*-*liquid chromatography*; ppm: parts per million; TFA: trifluoroacetic acid; V_*max*_: maximum velocity; WHO: World Health Organization; Z: carbobenzoxy.

## Supplementary material

The following online material is available for this article:

Additional file 1. (A) HPLC and mass spectrum reports of PEP1. (B) HPLC and mass spectrum reports of PEP2.

Additional file 2. Sequences found in PEAKS analysis (Figure 3A) present in the DPSA8 peptide.

Additional file 3. Sequences found in PEAKS analysis (Figure 3B) present in the DPKEN9 peptide.

Additional file 4. (A) Michaelis-Menten kinetics of elastase-1 and Abz-FRSSRQ-EDDnp (K_m_ was settled as 10.65 µM). (B) Michaelis-Menten kinetics of cathepsin L and Z-FR-MCA (K_m_ was settled as 2.65 µM).

## References

[B1] Chippaux JP (2017). Snakebite envenomation turns again into a neglected tropical
disease!. J Venom Anim Toxins incl Trop Dis.

[B2] Ferreira RS, de Barros LC, Abbade LPF, Barraviera SRCS, Silvares MRC, de Pontes LG (2017). Heterologous fibrin sealant derived from snake venom: from bench
to bedside - an overview. J Venom Anim Toxins incl Trop Dis.

[B3] Yao YT, Yuan X, Fang NX (2019). Hemocoagulase reduces postoperative bleeding and blood
transfusion in cardiac surgical patients: A PRISMA-compliant systematic
review and meta-analysis. Medicine (Baltimore).

[B4] Ondetti MA, Rubin B, Cushman DW (1977). Design of specific inhibitors of angiotensin-converting enzyme:
new class of orally active antihypertensive agents. Science.

[B5] Smith CG, Vane JR (2003). The discovery of captopril. FASEB J.

[B6] Závada J, Valenta J, Kopecký O, Stach Z, Leden P (2011). Black mamba dendroaspis polylepis bite: a case
report. Prague Med Rep.

[B7] Laustsen AH, Lomonte B, Lohse B, Fernández J, Gutiérrez JM (2015). Unveiling the nature of black mamba (Dendroaspis polylepis) venom
through venomics and antivenom immunoprofiling: Identification of key toxin
targets for antivenom development. J Proteomics.

[B8] Kunitz M (1945). Crystallization of a trypsin inhibitor from
soybean. Science.

[B9] Zhao R, Dai H, Qiu S, Li T, He Y, Ma Y (2011). SdPI, the first functionally characterized Kunitz-type trypsin
inhibitor from scorpion venom. PLoS One.

[B10] Mourão CB, Schwartz EF (2013). Protease inhibitors from marine venomous animals and their
counterparts in terrestrial venomous animals. Mar Drugs.

[B11] Ranasinghe S, McManus DP (2013). Structure and function of invertebrate Kunitz serine protease
inhibitors. Dev Comp Immunol.

[B12] Bendre AD, Ramasamy S, Suresh CG (2018). Analysis of Kunitz inhibitors from plants for comprehensive
structural and functional insights. Int J Biol Macromol.

[B13] Harvey AL, Robertson B (2004). Dendrotoxins: structure-activity relationships and effects on
potassium ion channels. Curr Med Chem.

[B14] ENG WS (2015). Kunitz Peptides. Venomous Reptiles and Their Toxins: Evolution, Pathophysiology, and
Biodiscovery.

[B15] Yang W, Feng J, Wang B, Cao Z, Li W, Wu Y (2014). BF9, the first functionally characterized snake toxin peptide
with Kunitz-type protease and potassium channel inhibiting
properties. J Biochem Mol Toxicol.

[B16] Neurath H (1999). Proteolytic enzymes, past and future. Proc Natl Acad Sci U S A.

[B17] Ceolin Mariano DO, de Oliveira Ú, Zaharenko AJ, Pimenta DC, Rádis-Baptista G, Prieto-da-Silva Á (2019). Bottom-up proteomic analysis of polypeptide venom components of
the giant ant. Toxins (Basel).

[B18] Portaro FC, Santos AB, Cezari MH, Juliano MA, Juliano L, Carmona E (2000). Probing the specificity of cysteine proteinases at subsites
remote from the active site: analysis of P4, P3, P2' and P3' variations in
extended substrates. Biochem J.

[B19] Smith PK, Krohn RI, Hermanson GT, Mallia AK, Gartner FH, Provenzano MD (1985). Measurement of protein using bicinchoninic acid. Anal Biochem.

[B20] Segel IH (1975). Enzyme Kinetics: Behavior and Analysis of Rapid Equilibrium and
Steady-State Enzyme Systems.

[B21] Pierce BG, Wiehe K, Hwang H, Kim BH, Vreven T, Weng Z (2014). ZDOCK server: interactive docking prediction of protein-protein
complexes and symmetric multimers. Bioinformatics.

[B22] Pierce B, Weng Z (2008). A combination of rescoring and refinement significantly improves
protein docking performance. Proteins.

[B23] Laskowski RA, Swindells MB (2011). LigPlot+: multiple ligand-protein interaction diagrams for drug
discovery. J Chem Inf Model.

[B24] Robertson B, Owen D, Stow J, Butler C, Newland C (1996). Novel effects of dendrotoxin homologues on subtypes of mammalian
Kv1 potassium channels expressed in Xenopus oocytes. FEBS Lett.

[B25] Rawlings ND, Barrett AJ, Thomas PD, Huang X, Bateman A, Finn RD (2018). he MEROPS database of proteolytic enzymes, their substrates and
inhibitors in 2017 and a comparison with peptidases in the PANTHER
database. Nucleic Acids Res.

[B26] World Health Organization (2010). Guidelines for the prevention and clinical management of snakebite in
Africa.

[B27] Glenn JL, Straight R (1978). Mojave rattlesnake Crotalus scutulatus scutulatus venom:
variation in toxicity with geographical origin. Toxicon.

[B28] Gonçalves-Machado L, Pla D, Sanz L, Jorge RJB, Leitão-De-Araújo M, Alves MLM (2016). Combined venomics, venom gland transcriptomics, bioactivities,
and antivenomics of two Bothrops jararaca populations from geographic
isolated regions within the Brazilian Atlantic rainforest. J Proteomics.

[B29] Lancelin JM, Foray MF, Poncin M, Hollecker M, Marion D (1994). Proteinase inhibitor homologues as potassium channel
blockers. Nat Struct Biol.

[B30] Smith D, Tikhonova IG, Jewhurst HL, Drysdale OC, Dvořák J, Robinson MW (2016). Unexpected activity of a novel kunitz-type inhibitor: inhibition
of cysteine proteases but not serine proteases. J Biol Chem.

[B31] Sasaki SD, Cotrin SS, Carmona AK, Tanaka AS (2006). An unexpected inhibitory activity of Kunitz-type serine
proteinase inhibitor derived from Boophilus microplus trypsin inhibitor on
cathepsin L. Biochem Biophys Res Commun.

[B32] Kolkenbrock H, Tschesche H (1987). A new inhibitor of elastase from the sea anemone (Anemonia
sulcata). Biol Chem Hoppe Seyler.

[B33] Shamsi TN, Parveen R, Amir M, Baig MA, Qureshi MI, Ali S (2016). Allium sativum protease inhibitor: A novel kunitz trypsin
inhibitor from garlic is a new comrade of the serpin family. PLoS One.

[B34] Chabbat J, Porte P, Tellier M, Steinbuch M (1993). Aprotinin is a competitive inhibitor of the factor VIIa-tissue
factor complex. Thromb Res.

[B35] Oddepally R, Sriram G, Guruprasad L (2013). Purification and characterization of a stable Kunitz trypsin
inhibitor from Trigonella foenum-graecum (fenugreek) seeds. Phytochemistry.

