# Supplementary material for: A Kunitz-type peptide from Dendroaspis polylepis venom as a simultaneous inhibitor of serine and cysteine proteases
Source: J Venom Anim Toxins Incl Trop Dis. 2020 Oct 7;26:e20200037. doi: 10.1590/1678-9199-JVATITD-2020-0037 (PMC7546081; doi:10.1590/1678-9199-JVATITD-2020-0037)

J Venom Anim Toxins incl Trop Dis,

https://doi.org/10.1590/1678-9199-JVATITD-2020-0037

Supplementary Material to "A Kunitz-type peptide from *Dendroaspis polylepis* venom as a simultaneous inhibitor of serine and cysteine proteases"

Additional file 1. (A) HPLC and mass spectrum reports of PEP1. **(B)** HPLC and mass spectrum reports of PEP2.

A

HPLC Report

Structure: KS-16
Sequence: [H]-KKQCEGFTWSGCGGNS-[OH]
Order ID/Name: 104268-1/Item-1
Lot No.: WB176136-P181221
Column: 4.6×250 mm, Kromasil 100-5C18
Solvent A: 0.1% trifluoroacetic in 100% acetonitrile
Solvent B: 0.1% trifluoroacetic in 100% water
Gradient:

	A	B
0.01min	15%	85%
25min	40%	60%
25.1min	100%	0%
30min	STOP	

Flow rate: 1.0 mL/min
Wavelength: 220 nm
Volume: 5 µl

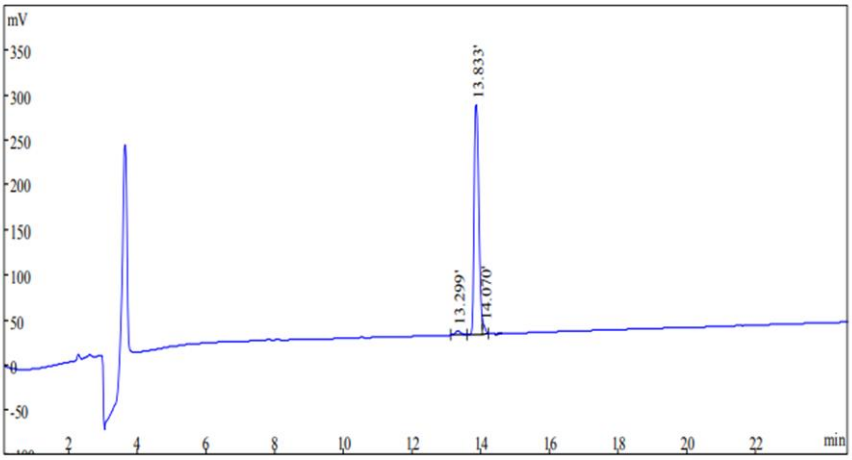

Peak No.	Ret Time	Height	Area	Conc.
1	13.299	4417	40261	1.885
2	13.833	254676	2054531	96.18
3	14.070	7883	41414	1.939
Total				100.0000

Mass Spectrum Report

Order ID: 104268-1
Sequence: [H]-KKQCEGFTWSGCGGNS-[OH]

Data Acquired: 1/5/2019
Acquired by: SHAO
Injection Volume: 1
Sample Name: 104268-1
MW: 1689.01
Lot No.: WB176136-P181221

Probe: ESI
Nebulizer Gas Floe: 1.5 L/min
CDL: -20.0 V
CDL Temp: 250 °C
Block Temp: 200 °C

Probe bias: 4.5 kV
Detector: 1.5 kV
T.Flow: 0.2 mL/min
B.conc: 50% H_2O/50% ACN

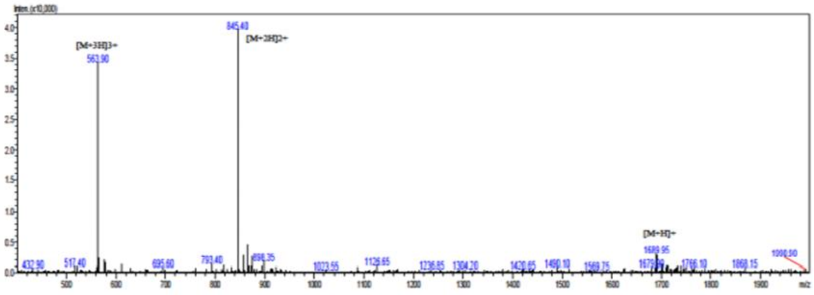

B

HPLC Report

Structure: PG-30
Sequence: [H]-PGRCYQKIPAFYYNQKKKQVEGFTWSGCGG-[OH]
Order ID/Name: 104268-2/Item-2
Lot No.: WB176137-P181221
Column: 4.6×250 mm, Kromasil 100-5C18
Solvent A: 0.1% trifluoroacetic in 100% acetonitrile
Solvent B: 0.1% trifluoroacetic in 100% water
Gradient:

	A	B
0.01min	26%	74%
25min	51%	49%
25.1min	100%	0%
30min	STOP	

Flow rate: 1.0 mL/min
Wavelength: 220 nm
Volume: 5 µl

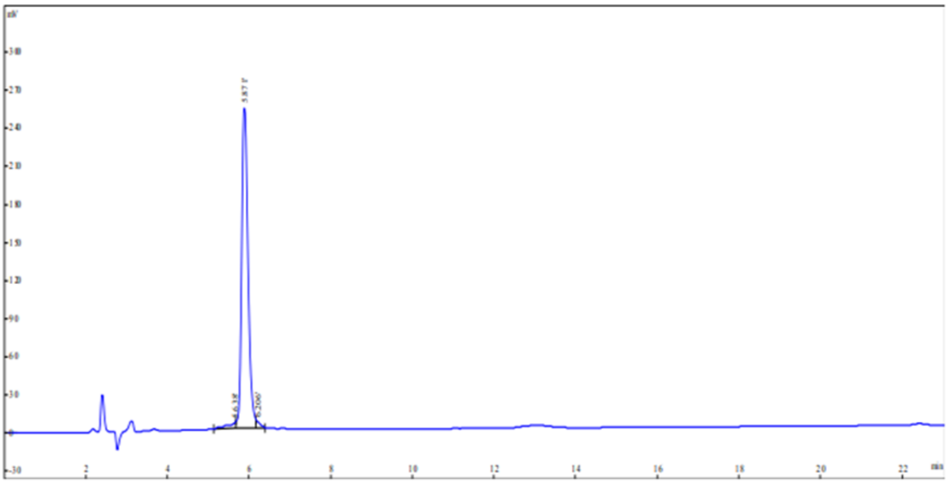

Peak No.	Ret Time	Height	Area	Conc.
1	5.638	4258	72307	2.787
2	5.871	251710	2481895	96.03
3	6.206	4331	30632	1.185
Total				100.0000

Mass Spectrum Report

Order ID: 104268-2
Sequence: [H]-PGRCYQKIPAFYYNQKKKQVEGFTWSGCGG-[OH]

Data Acquired: 1/5/2019
Acquired by: YU
Injection Volume: 1
Sample Name: 104268-2
MW: 3439.95
Lot No.: WB176137-P181221

Probe: ESI
Nebulizer Gas Floe: 1.5 L/min
CDL: -20.0 V
CDL Temp: 250 °C
Block Temp: 200 °C

Probe bias: 4.5 kV
Detector: 1.5 kV
T.Flow: 0.2 mL/min
B.conc: 50% H_2O/50% ACN

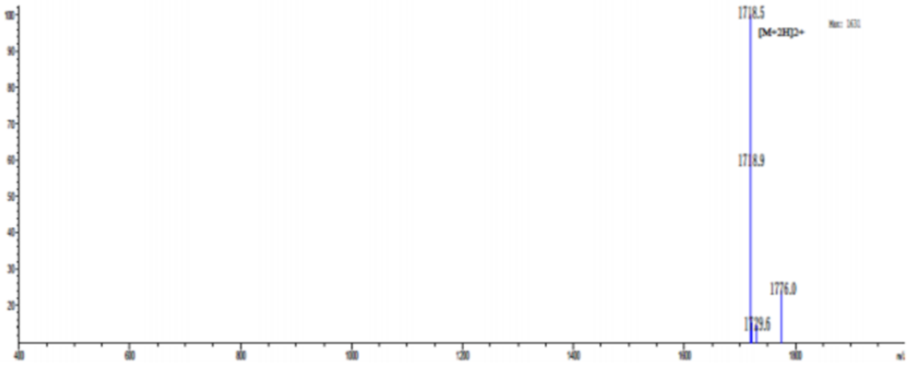

Supplement: Additional file 1. [file 1678-9199-jvatitd-26-e20200037-s1.pdf]
