# Supplementary material for: A Kunitz-type peptide from Dendroaspis polylepis venom as a simultaneous inhibitor of serine and cysteine proteases
Source: J Venom Anim Toxins Incl Trop Dis. 2020 Oct 7;26:e20200037. doi: 10.1590/1678-9199-JVATITD-2020-0037 (PMC7546081; doi:10.1590/1678-9199-JVATITD-2020-0037)
Supplement: Additional file 2. [file 1678-9199-jvatitd-26-e20200037-s2.pdf]

J Venom Anim Toxins incl Trop Dis,

https://doi.org/10.1590/1678-9199-JVATITD-2020-0037

Supplementary Material to "A Kunitz-type peptide from *Dendroaspis polylepis* venom as a simultaneous inhibitor of serine and cysteine proteases"

Additional file 2: Sequences found in Peaks analysis (Figure 3A) present in the DPSA8 peptide.

Peptide	-10lgP	Mass	Length	ppm	m/z	Z	RT	Accession	PTM
**KQC(+57.02)EGFTWSGC(+57.02)GGNSNR**	89.82	1943.8003	17	0.2	972.9077	2	18.08	sp|P00979|	Carbamidomethylation
**KQC(+57.02)EGFTWSGC(+57.02)GGNSNR**	54.88	1943.8003	17	0.4	648.9410	3	18.08	sp|P00979|	Carbamidomethylation
**KKQC(+57.02)EGFTWSGC(+57.02)GGNSNR**	86.50	2071.8953	18	0.8	1036.9557	2	17.60	sp|P00979|	Carbamidomethylation
**KKQC(+57.02)EGFTWSGC(+57.02)GGNSNR**	57.10	2071.8953	18	-0.5	691.6387	3	17.59	sp|P00979|	Carbamidomethylation
**KKQC(+57.02)EGFTWSGC(+57.02)GGNSNR**	19.87	2071.8953	18	-0.5	518.9808	4	17.63	sp|P00979|	Carbamidomethylation
**QC(+57.02)EGFTWSGC(+57.02)GGNSNR**	74.85	1815.7053	16	-0.3	908.8597	2	18.72	sp|P00979|	Carbamidomethylation
**QC(+57.02)EGFTWSGC(+57.02)GGNSNR**	20.73	1815.7053	16	0.2	908.8601	2	19.54	sp|P00979|	Carbamidomethylation
**KQC(+57.02)EGFTWSGC(+57.02)GGNSNRFK**	68.73	2218.9636	19	-0.2	1110.4889	2	18.25	sp|P00979|	Carbamidomethylation
**KQC(+57.02)EGFTWSGC(+57.02)GGNSNRFK**	53.69	2218.9636	19	-0.3	740.6616	3	18.23	sp|P00979|	Carbamidomethylation
**KQC(+57.02)EGFTWSGC(+57.02)GGNSNRFK**	16.82	2218.9636	19	-0.1	555.7481	4	18.24	sp|P00979|	Carbamidomethylation
**QC(+57.02)EGFTWSGC(+57.02)GGNSNRFK**	67.03	2090.8687	18	-0.1	1046.4415	2	18.86	sp|P00979|	Carbamidomethylation
**C(+57.02)YQKIPAFYYNQK**	66.88	1721.8235	13	0.4	861.9194	2	18.86	sp|P00979|	Carbamidomethylation
**C(+57.02)YQKIPAFYYNQK**	62.17	1721.8235	13	0.3	574.9486	3	18.88	sp|P00979|	Carbamidomethylation
**C(+57.02)YQKIPAFYYNQK**	38.79	1721.8235	13	-0.2	574.9483	3	19.15	sp|P00979|	Carbamidomethylation
**IPAFYYNQKK**	63.70	1270.6709	10	0.1	636.3428	2	18.36	sp|P00979|	
**IPAFYYNQKK**	18.02	1270.6709	10	0.8	424.5646	3	18.54	sp|P00979|	
**IPAFYYNQK**	61.01	1142.5760	9	1.6	572.2962	2	19.07	sp|P00979|	
**IPAFYYNQK**	56.09	1142.5760	9	-0.7	572.2949	2	19.60	sp|P00979|	
**IPAFYYNQK**	54.25	1142.5760	9	0.0	572.2953	2	19.33	sp|P00979|	
**IPAFYYNQK**	51.00	1142.5760	9	-1.9	572.2942	2	19.86	sp|P00979|	
**IPAFYYNQK**	44.16	1142.5760	9	0.6	572.2957	2	20.43	sp|P00979|	
**C(+57.02)YQKIPAFYYNQKK**	52.59	1849.9185	14	-0.1	617.6467	3	18.32	sp|P00979|	Carbamidomethylation

Peptide	-10lgP	Mass	Length	ppm	m/z	Z	RT	Accession	PTM
**C(+57.02)YQKIPAFYYNQKK**	49.29	1849.9185	14	-0.1	925.9664	2	18.34	sp|P00979|	Carbamidomethylation
**TWSGC(+57.02)GGNSNRFK**	49.26	1469.6470	13	-0.5	735.8304	2	17.10	sp|P00979|	Carbamidomethylation
**KKQC(+57.02)EGFTWSGC(+57.02)GGN**	47.31	1714.7191	15	-0.7	858.3663	2	18.33	sp|P00979|	Carbamidomethylation
**SGC(+57.02)GGNSNRFK**	46.66	1182.5200	11	-1.1	592.2667	2	15.15	sp|P00979|	Carbamidomethylation
**SGC(+57.02)GGNSNRFK**	38.33	1182.5200	11	-0.2	592.2672	2	15.40	sp|P00979|	Carbamidomethylation
**TWSGC(+57.02)GGNSNR**	41.60	1194.4836	11	-0.4	598.2488	2	16.23	sp|P00979|	Carbamidomethylation
**C(+57.02)YQKIPAFYYN**	39.03	1465.6700	11	-0.8	733.8417	2	19.64	sp|P00979|	Carbamidomethylation
**C(+57.02)YQKIPAFY**	38.46	1188.5637	9	-0.2	595.2890	2	19.66	sp|P00979|	Carbamidomethylation
**C(+57.02)YQKIPAFYY**	37.43	1351.6271	10	-1.6	676.8197	2	19.94	sp|P00979|	Carbamidomethylation
**KKQC(+57.02)EGFTW**	35.31	1182.5492	9	0.0	592.2819	2	19.14	sp|P00979|	Carbamidomethylation
**KKQC(+57.02)EGFTW**	28.37	1182.5492	9	-0.7	592.2814	2	19.40	sp|P00979|	Carbamidomethylation
**KLC(+57.02)ILHR**	34.86	938.5484	7	-0.9	470.2810	2	17.24	sp|P00979|	Carbamidomethylation
**KLC(+57.02)ILHR**	29.93	938.5484	7	0.2	470.2816	2	17.50	sp|P00979|	Carbamidomethylation
**KLC(+57.02)ILHR**	20.23	938.5484	7	0.2	313.8568	3	17.26	sp|P00979|	Carbamidomethylation
**FKTIEEC(+57.02)R**	34.66	1081.5226	8	0.2	361.5149	3	16.92	sp|P00979|	Carbamidomethylation
**FKTIEEC(+57.02)R**	31.42	1081.5226	8	-0.7	541.7682	2	16.92	sp|P00979|	Carbamidomethylation
**C(+57.02)YQKIPAF**	33.91	1025.5004	8	-1.2	513.7568	2	19.48	sp|P00979|	Carbamidomethylation
**QKIPAFYYNQK**	33.56	1398.7295	11	0.0	700.3720	2	18.52	sp|P00979|	
**QKIPAFYYNQK**	23.74	1398.7295	11	-0.1	467.2504	3	18.63	sp|P00979|	
**KQC(+57.02)EGFTW**	33.33	1054.4542	8	-1.4	528.2336	2	19.68	sp|P00979|	Carbamidomethylation
**KKQC(+57.02)EGF**	29.42	895.4222	7	0.0	448.7184	2	16.05	sp|P00979|	Carbamidomethylation
**TIEEC(+57.02)R**	29.06	806.3593	6	0.0	404.1869	2	15.74	sp|P00979|	Carbamidomethylation
**LC(+57.02)ILHR**	27.95	810.4534	6	0.2	406.2341	2	17.89	sp|P00979|	Carbamidomethylation
**LC(+57.02)ILHR**	18.12	810.4534	6	0.3	271.1585	3	17.90	sp|P00979|	Carbamidomethylation
**FKTIEEC(+57.02)RR**	27.41	1237.6237	9	-0.5	619.8188	2	16.31	sp|P00979|	Carbamidomethylation
**FKTIEEC(+57.02)RR**	21.75	1237.6237	9	0.5	619.8194	2	16.57	sp|P00979|	Carbamidomethylation
**TIEEC(+57.02)RR**	21.17	962.4603	7	0.3	482.2376	2	15.65	sp|P00979|	Carbamidomethylation
**TIEEC(+57.02)RR**	19.08	962.4603	7	0.0	482.2374	2	15.93	sp|P00979|	Carbamidomethylation
**RTC(+57.02)IR**	20.91	704.3752	5	0.5	353.1950	2	14.37	sp|P00979|	Carbamidomethylation
**TC(+57.02)IRK**	18.76	676.3690	5	0.5	339.1920	2	14.09	sp|P00979|	Carbamidomethylation

Peptide	-10lgP	Mass	Length	ppm	m/z	Z	RT	Accession	PTM
**TC(+57.02)IRK**	18.29	676.3690	5	0.5	339.1920	2	13.54	sp|P00979|	Carbamidomethylation
**TC(+57.02)IRK**	17.57	676.3690	5	0.3	339.1919	2	14.36	sp|P00979|	Carbamidomethylation
**TC(+57.02)IRK**	16.49	676.3690	5	0.3	339.1919	2	13.82	sp|P00979|	Carbamidomethylation
**KQC(+57.02)EGF**	18.64	767.3272	6	0.0	384.6709	2	17.06	sp|P00979|	Carbamidomethylation
**C(+57.02)YQKIPA**	15.81	878.4320	7	0.0	440.2233	2	17.27	sp|P00979|	Carbamidomethylation
**QC(+57.02)EGFTW**	15.19	926.3593	7	-0.3	464.1868	2	20.37	sp|P00979|	Carbamidomethylation

-10logP = peptide score; RT = Retention time; m/z = mass/charge; Z = charge; PTM = Post-translational modification
