# Supplementary material for: A Kunitz-type peptide from Dendroaspis polylepis venom as a simultaneous inhibitor of serine and cysteine proteases
Source: J Venom Anim Toxins Incl Trop Dis. 2020 Oct 7;26:e20200037. doi: 10.1590/1678-9199-JVATITD-2020-0037 (PMC7546081; doi:10.1590/1678-9199-JVATITD-2020-0037)
Supplement: Additional file 3. [file 1678-9199-jvatitd-26-e20200037-s3.pdf]

J Venom Anim Toxins incl Trop Dis,

https://doi.org/10.1590/1678-9199-JVATITD-2020-0037

Supplementary Material to "A Kunitz-type peptide from *Dendroaspis polylepis* venom as a simultaneous inhibitor of serine and cysteine proteases"

Additional file 3: Sequences found in Peaks analysis (Figure 3B) present in the DPKEN9 peptide.

Peptide	-10lgP	Mass	Length	ppm	m/z	Z	RT	Accession	PTM
**KQC(+57.02)EGFTWSGC(+57.02)GGNSNR**	85.37	1943.8003	17	-0.1	972.9073	2	18.28	sp|P00979|	Carbamidomethylation
**KQC(+57.02)EGFTWSGC(+57.02)GGNSNR**	47.24	1943.8003	17	-0.1	648.9407	3	18.28	sp|P00979|	Carbamidomethylation
**QC(+57.02)EGFTWSGC(+57.02)GGNSNR**	66.08	1815.7053	16	0.0	908.8599	2	18.82	sp|P00979|	Carbamidomethylation
**QC(+57.02)EGFTWSGC(+57.02)GGNSNR**	30.57	1815.7053	16	-0.3	606.2422	3	19.02	sp|P00979|	Carbamidomethylation
**QC(+57.02)EGFTWSGC(+57.02)GGNSNR**	19.89	1815.7053	16	-0.6	908.8594	2	19.55	sp|P00979|	Carbamidomethylation
**C(+57.02)YQKIPAFYYNQKK**	63.34	1849.9185	14	-0.3	617.6466	3	18.53	sp|P00979|	Carbamidomethylation
**C(+57.02)YQKIPAFYYNQKK**	61.35	1849.9185	14	0.4	925.9669	2	18.58	sp|P00979|	Carbamidomethylation
**KKQC(+57.02)EGFTWSGC(+57.02)GGNSNR**	63.19	2071.8953	18	0.3	1036.9552	2	17.82	sp|P00979|	Carbamidomethylation
**KKQC(+57.02)EGFTWSGC(+57.02)GGNSNR**	45.57	2071.8953	18	-1.0	691.6383	3	17.80	sp|P00979|	Carbamidomethylation
**C(+57.02)YQKIPAFYYNQK**	58.74	1721.8235	13	0.6	861.9196	2	19.06	sp|P00979|	Carbamidomethylation
**C(+57.02)YQKIPAFYYNQK**	55.41	1721.8235	13	-0.6	574.9481	3	19.04	sp|P00979|	Carbamidomethylation
**IPAFYYNQKK**	56.58	1270.6709	10	0.7	636.3432	2	18.55	sp|P00979|	
**IPAFYYNQKK**	28.02	1270.6709	10	1.3	424.5648	3	18.55	sp|P00979|	
**IPAFYYNQK**	54.77	1142.5760	9	0.8	572.2958	2	19.17	sp|P00979|	
**IPAFYYNQK**	54.21	1142.5760	9	-0.3	572.2951	2	19.45	sp|P00979|	
**IPAFYYNQK**	45.24	1142.5760	9	-1.3	572.2946	2	19.72	sp|P00979|	
**IPAFYYNQK**	45.15	1142.5760	9	0.2	572.2954	2	21.82	sp|P00979|	
**IPAFYYNQK**	43.59	1142.5760	9	-1.3	572.2946	2	20.50	sp|P00979|	
**IPAFYYNQK**	42.06	1142.5760	9	-0.3	572.2951	2	21.02	sp|P00979|	
**IPAFYYNQK**	41.78	1142.5760	9	-0.6	572.2950	2	19.99	sp|P00979|	
**IPAFYYNQK**	41.61	1142.5760	9	0.1	572.2953	2	20.77	sp|P00979|	

Peptide	-10lgP	Mass	Length	ppm	m/z	Z	RT	Accession	PTM
**IPAFYYNQK**	39.13	1142.5760	9	-0.1	572.2952	2	22.77	sp|P00979|	
**IPAFYYNQK**	38.83	1142.5760	9	-1.0	572.2947	2	20.23	sp|P00979|	
**IPAFYYNQK**	38.45	1142.5760	9	0.3	572.2955	2	22.07	sp|P00979|	
**IPAFYYNQK**	37.76	1142.5760	9	0.3	572.2955	2	21.56	sp|P00979|	
**IPAFYYNQK**	34.10	1142.5760	9	-0.3	572.2951	2	22.35	sp|P00979|	
**IPAFYYNQK**	33.16	1142.5760	9	0.0	572.2953	2	21.31	sp|P00979|	
**TWSGC(+57.02)GGNSNRFK**	45.24	1469.6470	13	-1.2	735.8299	2	17.36	sp|P00979|	Carbamidomethylation
**SGC(+57.02)GGNSNRFK**	43.79	1182.5200	11	-1.6	592.2664	2	15.48	sp|P00979|	Carbamidomethylation
**KQC(+57.02)EGFTWSGC(+57.02)GGNSNRFK**	39.54	2218.9636	19	-0.5	740.6614	3	18.48	sp|P00979|	Carbamidomethylation
**C(+57.02)YQKIPAFYY**	35.00	1351.6271	10	-0.3	676.8206	2	20.03	sp|P00979|	Carbamidomethylation
**FKTIEEC(+57.02)R**	33.93	1081.5226	8	-0.4	541.7684	2	17.18	sp|P00979|	Carbamidomethylation
**FKTIEEC(+57.02)R**	29.76	1081.5226	8	0.5	361.5150	3	17.19	sp|P00979|	Carbamidomethylation
**KLC(+57.02)ILHR**	32.74	938.5484	7	-0.5	470.2812	2	17.57	sp|P00979|	Carbamidomethylation
**KLC(+57.02)ILHR**	29.86	938.5484	7	-0.2	470.2814	2	17.85	sp|P00979|	Carbamidomethylation
**KLC(+57.02)ILHR**	18.38	938.5484	7	0.1	313.8568	3	17.57	sp|P00979|	Carbamidomethylation
**KQC(+57.02)EGFTW**	32.69	1054.4542	8	-0.6	528.2341	2	19.80	sp|P00979|	Carbamidomethylation
**QKIPAFYYNQK**	31.40	1398.7295	11	0.1	700.3721	2	18.66	sp|P00979|	
**QKIPAFYYNQK**	23.73	1398.7295	11	-0.6	467.2502	3	18.75	sp|P00979|	
**LC(+57.02)ILHR**	29.94	810.4534	6	0.7	406.2343	2	18.10	sp|P00979|	Carbamidomethylation
**LC(+57.02)ILHR**	26.76	810.4534	6	0.1	406.2340	2	18.37	sp|P00979|	Carbamidomethylation
**LC(+57.02)ILHR**	24.91	810.4534	6	0.6	406.2342	2	18.65	sp|P00979|	Carbamidomethylation
**LC(+57.02)ILHR**	19.58	810.4534	6	0.6	271.1586	3	18.10	sp|P00979|	Carbamidomethylation
**LC(+57.02)ILHR**	19.26	810.4534	6	0.0	406.2340	2	19.03	sp|P00979|	Carbamidomethylation
**TIEEC(+57.02)R**	27.76	806.3593	6	0.4	404.1871	2	15.92	sp|P00979|	Carbamidomethylation
**TIEEC(+57.02)RR**	26.30	962.4603	7	0.0	482.2375	2	16.12	sp|P00979|	Carbamidomethylation
**TIEEC(+57.02)RR**	19.79	962.4603	7	-0.1	482.2374	2	15.83	sp|P00979|	Carbamidomethylation
**TIEEC(+57.02)RR**	17.67	962.4603	7	0.2	482.2375	2	15.30	sp|P00979|	Carbamidomethylation
**TIEEC(+57.02)RR**	16.66	962.4603	7	-0.7	482.2371	2	15.57	sp|P00979|	Carbamidomethylation
**KKQC(+57.02)EGF**	25.96	895.4222	7	0.1	448.7184	2	16.39	sp|P00979|	Carbamidomethylation
**KKQC(+57.02)EGF**	22.74	895.4222	7	-0.3	448.7182	2	16.65	sp|P00979|	Carbamidomethylation

Peptide	-10lgP	Mass	Length	ppm	m/z	Z	RT	Accession	PTM
**FKTIEEC(+57.02)RR**	24.27	1237.6237	9	0.1	619.8192	2	16.64	sp|P00979|	Carbamidomethylation
**TWSGC(+57.02)GGNSNR**	23.01	1194.4836	11	-0.3	598.2489	2	16.43	sp|P00979|	Carbamidomethylation
**KKQC(+57.02)EGFTW**	22.98	1182.5492	9	-0.7	592.2814	2	19.33	sp|P00979|	Carbamidomethylation
**RTC(+57.02)IR**	21.83	704.3752	5	0.6	353.1951	2	14.87	sp|P00979|	Carbamidomethylation
**RTC(+57.02)IR**	16.18	704.3752	5	0.2	353.1949	2	14.60	sp|P00979|	Carbamidomethylation
**QC(+57.02)EGFTWSGC(+57.02)GGNSNRFK**	21.04	2090.8687	18	-0.4	697.9632	3	18.99	sp|P00979|	Carbamidomethylation
**KQC(+57.02)EGF**	18.17	767.3272	6	-0.7	384.6706	2	17.32	sp|P00979|	Carbamidomethylation
**TC(+57.02)IRK**	17.16	676.3690	5	0.2	339.1919	2	15.70	sp|P00979|	Carbamidomethylation
**TC(+57.02)IRK**	16.63	676.3690	5	0.4	339.1919	2	14.21	sp|P00979|	Carbamidomethylation

-10logP = peptide score; RT = Retention time; m/z = mass/charge; Z = charge; PTM = Post-translational modification
