# Supplementary material for: A Kunitz-type peptide from Dendroaspis polylepis venom as a simultaneous inhibitor of serine and cysteine proteases
Source: J Venom Anim Toxins Incl Trop Dis. 2020 Oct 7;26:e20200037. doi: 10.1590/1678-9199-JVATITD-2020-0037 (PMC7546081; doi:10.1590/1678-9199-JVATITD-2020-0037)
Supplement: Additional file 4. [file 1678-9199-jvatitd-26-e20200037-s4.pdf]

J Venom Anim Toxins incl Trop Dis,

https://doi.org/10.1590/1678-9199-JVATITD-2020-0037

Supplementary Material to "A Kunitz-type peptide from *Dendroaspis polylepis* venom as a simultaneous inhibitor of serine and cysteine proteases"

Additional file 4. (A) Michaelis-Menten kinetics of elastase-1 and Abz-FRSSRQ-EDDnp (K_m was settled as 10.65 µM). **(B)** Michaelis-Menten kinetics of cathepsin L and Z-FR-MCA (K_m was settled as 2.65 µM). The experimental procedures are described in the "Methods" section.

A

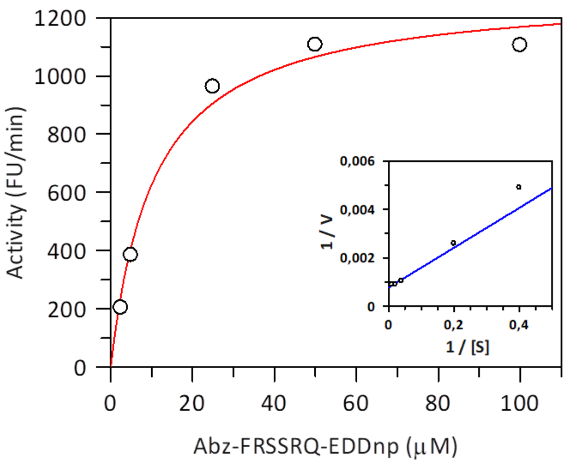

Parameter	Value	Std. Error
Vmax	1292.5232	71.2661
Km	10.6498	2.2721

B

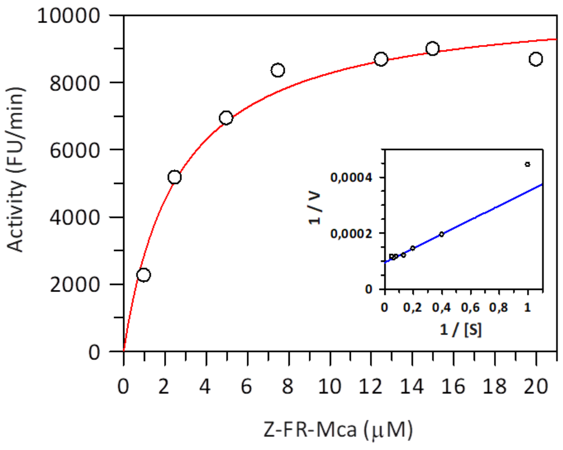

Parameter	Value	Std. Error
Vmax	10462.3469	502.0623
Km	2.6527	0.4867
